# Commissioning an Exradin W2 plastic scintillation detector for clinical use in small radiation fields

**DOI:** 10.1002/acm2.13728

**Published:** 2022-07-21

**Authors:** Dustin J. Jacqmin, Jessica R. Miller, Brendan A. Barraclough, Zacariah E. Labby

**Affiliations:** ^1^ Department of Human Oncology University of Wisconsin‐Madison Madison Wisconsin USA; ^2^ Department of Medical Physics University of Wisconsin‐Madison Madison Wisconsin USA; ^3^ Department of Radiation Oncology Duke University Medical Center Durham North Carolina USA

**Keywords:** Exradin W2, plastic scintillation detector, radiation measurement, small field dosimetry, stereotactic radiosurgery

## Abstract

**Purpose:**

The purpose of this work is to evaluate the Standard Imaging Exradin W2 plastic scintillation detector (W2) for use in the types of fields used for stereotactic radiosurgery.

**Methods:**

Prior to testing the W2 in small fields, the W2 was evaluated in standard large field conditions to ensure good detector performance. These tests included energy dependence, short‐term repeatability, dose‐response linearity, angular dependence, temperature dependence, and dose rate dependence. Next, scan settings and calibration of the W2 were optimized to ensure high quality data acquisition. Profiles of small fields shaped by cones and multi‐leaf collimator (MLCs) were measured using the W2 and IBA RAZOR diode in a scanning water tank. Output factors for cones (4–17.5 mm) and MLC fields (1, 2, 3 cm) were acquired with both detectors. Finally, the dose at isocenter for seven radiosurgery plans was measured with the W2 detector.

**Results:**

W2 exhibited acceptable warm‐up behavior, short‐term reproducibility, axial angular dependence, dose‐rate linearity, and dose linearity. The detector exhibits a dependence upon energy, polar angle, and temperature. Scanning measurements taken with the W2 and RAZOR were in good agreement, with full‐width half‐maximum and penumbra widths agreeing to within 0.1 mm. The output factors measured by the W2 and RAZOR exhibited a maximum difference of 1.8%. For the seven point‐dose measurements of radiosurgery plans, the W2 agreed well with our treatment planning system with a maximum deviation of 2.2%. The Čerenkov light ratio calibration method did not significantly impact the measurement of relative profiles, output factors, or point dose measurements.

**Conclusion:**

The W2 demonstrated dosimetric characteristics that are suitable for radiosurgery field measurements. The detector agreed well with the RAZOR diode for output factors and scanned profiles and showed good agreement with the treatment planning system in measurements of clinical treatment plans.

## 1 INTRODUCTION

The plastic scintillation detector has been shown to be a promising water‐equivalent detector for small and nonstandard fields.^1^, ^2^, ^3^, ^4^ In 2013, the first commercial scintillator detector, the Exradin W1 (Standard Imaging, Middleton, WI), was released. The detector, which has 1 × 3 mm active element, has been well characterized in the literature.^5^, ^6^, ^7^, ^8^, ^9^, ^10^, ^11^ A newer version of this device, the Exradin W2 scintillator detector was released in 2018. The W2 detector set includes 1 × 1 and 1 × 3 mm detectors and can be used in scanning mode unlike its predecessor. The detectors are provided with an optimized electrometer system, discussed in Galavis et al.,^12^ which maintains a higher signal‐to‐noise ratio than the W1 detector system.

The W1 scintillator was thoroughly characterized by Carassco et al. in 2014. Characterization of the detector included absolute dose measurements, energy dependence, short‐term repeatability, dose‐response linearity, angular dependence, temperature dependence, dose‐per‐pulse dependence, and monitor units (MU) repetition rate dependence.^6^ Recently, Galavis et al. performed a subset of these measurements for the W2 scintillator.^12^ In addition, Rudek et al. compared profile measurements between film and the W2 scintillator for a Gamma Knife system. The authors concluded that the W2 scintillator was an adequate replacement for film‐based profile tests.^13^

This work has three aims. First, we characterize the 1 × 1 mm W2 scintillator using the same tests performed for the W1 by Carassco et al., providing a direct comparison to this prior work and full set of commissioning data for the W2 scintillator. Second, we explore optimal use of the detector for linac‐based water‐tank measurements by investigating the effect of detector settings, scan settings, and Čerenkov light ratio (CLR) on measurements. Finally, we use the detector to collect data typically required for small radiation field modeling, including relative output factors, relative profiles, and point dose measurements for composite treatment plans.

## 2 METHODS

All measurements were performed with an Exradin W2 detector with a 1 × 1 mm scintillating element on a Varian TrueBeam linear accelerator (Varian Medical Systems, Palo Alto, CA).

### 2.1 LR

In addition to scintillation light produced in the active volume of the W2, radiation exposure also produces Čerenkov light in the active volume of the detector and the optical fiber. The correction method for the presence of Čerenkov light in the W1 and W2 plastic scintillation detector system is called the spectral method and has been discussed in detail in the literature.^11^, ^14^, ^15^, ^16^ Briefly, one must measure a CLR, which is used to correct raw measurements by eliminating the Čerenkov component of the signal. The measurement of CLR involves taking two measurements that result in an identical dose to the active volume but with different amounts of a detector fiber in the field. There is one measurement for a minimum fiber length configuration and one for a maximum length configuration. The CLR is given by the following formula:

(1) $$\mathrm{CLR}\;=\frac{R_{\mathrm{Blue},\max}-R_{\mathrm{Blue},\;\min}}{R_{\mathrm{Green},\max}-R_{\mathrm{Green},\min}}$$

Where *R*_Blue,max_ and *R*_Blue,min_ are the readings from the blue color channel in the maximum and minimum length configurations, respectively. Likewise, *R*_Green,max_ and *R*_Green,min_ are the readings from the green color channel in the maximum and minimum length configurations, respectively. Once the CLR has been determined, a dose conversion factor called the Gain (*G*) can be calculated as follows using an additional measurement in a reference field:

(2) $$G\;=\frac{D_{\mathrm{ref}}}{R_{\mathrm{Blue}}-\left(R_{Green}\;\times \;\mathrm{CLR}\right)}$$

Where *D*_ref_ is the dose to the active element of the scintillator in the reference field, and *R*_Blue_ and *R*_Green_ are the raw readings from the blue and green color channels in the reference field irradiation. With the CLR and Gain determined, one can calculate dose from raw readings using the following formula:

(3) $$M\;=\;G\times \left[R_{\mathrm{Blue}}-\left(R_{\mathrm{Green}}\times \mathrm{CLR}\right)\right]$$

Where *M* is the measured dose (corrected for the Čerenkov light effect), *G* and *CLR* are the Gain and CLR, and *R*_Blue_ and *R*_Green_ are the raw readings from the blue and green color channels.

**FIGURE 1:**
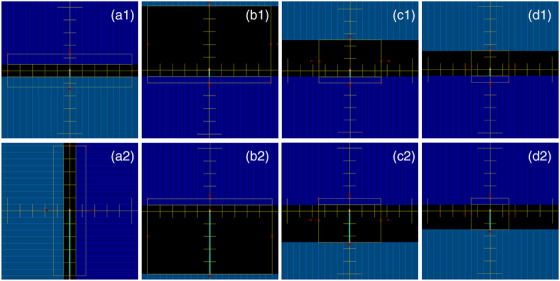
Radiation fields used to measure Čerenkov light ratio. The top row shows the minimum fiber length configurations, while the bottom row shows the maximum fiber length configurations. (a) 10 × 1 cm rectangle method, (b) 5.5 × 10 cm rectangle method, (c) 3 × 5 cm rectangle method, (d) 2 × 3 cm rectangle method. Jaw positions are shown with a faint yellow rectangle; MLC positions are displayed as opaque blue rectangles

We investigated four CLR measurement techniques. The first technique is one recommended in the vendor‐provided instruction manual. In the manual, it is called the “Rectangular field rotation method,” but we will call it the “10 × 1 cm rectangle method.” This technique uses a 10 × 1 cm field shaped by the multi‐leaf collimator (MLC) and centered on the central axis. In the minimum configuration (Figure 1A.1), the rectangle is oriented with the long axis of the rectangle perpendicular to the detector such that 0.5 cm of the detector and fiber are in the field. In the maximum configuration (Figure 1A.2), the rectangle is oriented with the long axis of the rectangle parallel to the detector such that 5 cm of detector and fiber are in the field. The two configurations use the same aperture and differ only in a 90‐degree rotation of the collimator.

We will call the second CLR technique the “5.5 × 10 cm rectangle method.” This technique uses a 5.5 × 10 cm MLC‐shaped field that is asymmetric relative to the central axis. To generate this shape, one can start with a 10 × 10 cm MLC‐shaped field, then move one of the banks of MLCs inward so that they are 0.5 cm from the central axis to generate a 5.5 × 10 cm field. In the minimum configuration (Figure 1B.1), the rectangle is oriented such that the shortened side is blocking all but 0.5 cm of detector fiber. In the maximum configuration (Figure 1B.2), the rectangle is rotated via a 180‐degree collimator rotation so that 5 cm of fiber is exposed. The third and fourth techniques (“3 × 5 cm rectangle method” in Figure 1C and “2 × 3 cm rectangle method” in Figure 1D) have similar designs to the second technique, but with smaller field sizes. Our custom techniques have 0.5 cm of fiber exposed in the minimum orientation; the amount of fiber exposed in the maximum orientation differs in each technique.

The motivation for investigating different CLR techniques was two‐fold. First, we found the “10 × 1 cm rectangle method” difficult to implement in a water tank. In the maximum configuration, the cable must be placed precisely in the middle of the 1‐cm wide rectangle to measure the CLR reproducibly, which is difficult to do in a water phantom. The custom methods are more robust to small changes in cable position. Second, given that the primary application of the W2 at our institution will be small field measurements, we wanted to determine if there were benefits to using smaller field shapes for CLR determination, thereby minimizing the differences between the calibration and measurement geometries. This motivated our choice of the 3 × 5 cm and 2 × 3 cm rectangles.

### 2.2 General characterization

The general characterization work required several different detector and beam geometries. The most common geometry was what we call the “reference‐like setup” due to the similarity to reference conditions used for calibration. The W2 was placed perpendicular to the central axis of the beam with the center of the detector at a depth of 10 cm in an IBA Blue Phantom 2 water tank (IBA Dosimetry, Schwarzenbruck, Germany). A 10 × 10 cm field was used with a source‐to‐surface distance (SSD) of 100 cm. The CLR and Gain values measured with the “5.5 × 10 cm rectangle method” were used for all general characterization measurements.

Multiple photon energies were used during testing. All tests used the 6 MV flattening filter‐free (FFF) beam because it is the primary energy used for radiosurgery and small targets at our institution. We also used 6 MV in tests where we believed dose rate may have an impact on the test (short‐term repeatability, dose‐response linearity, MU repetition rate, dose‐per‐pulse dependence). Four energies (6 MV, 6 MV FFF, 10 MV, and 10 MV FFF) were investigated for energy dependence.

#### 2.2.1 Energy dependence

The variation in response of the detector for 6 MV, 6 MV FFF, 10 MV, and 10 MV FFF was measured in the reference‐like setup. First, we measured dose in reference conditions for all energies using an NIST‐tracible Farmer‐type ionization chamber (Exradin A12, Standard Imaging, Middleton, WI). Then the W2 was calibrated for each energy using the dose measured with the ion chamber, which yielded a unique pair of CLR and Gain values for each energy.

Next, we configured the W2 software to apply the 6 MV FFF CLR and Gain values and measured dose under reference conditions for all four photon energies, three dose readings each. The average and standard deviation of the dose was calculated for each energy. Finally, we computed the percent difference between the dose measured with the W2 scintillator calibrated at 6 MV FFF and the dose measured with the ion chamber.

#### 2.2.2 Detector settling behavior

The detector settling behavior of the W2 was measured in the reference‐like setup using the 6 MV FFF beam. The W2 electrometer was powered on, and measurements of 50 MU began immediately. Raw readings from the blue and green channels were collected at intervals of roughly 9 s for 15 min. We computed the relative deviation over the 15‐min interval with respect to the mean readings for each color channel (blue and green). In addition, we qualitatively reviewed the time series to look for trends.

#### 2.2.3 Short‐term repeatability

Short‐term repeatability was measured for the 6 MV and 6 MV FFF beams in the reference‐like setup after the system was powered on for 15 min. A series of 20 measurements were performed for each repeatability study using manual data acquisition for 20 MU and 100 MU (approximately 0.13 and 0.65 Gy). The 100 MU measurements were repeated with triggered data collection to compare triggered versus manual data collection. The standard deviation of each measurement set was calculated to quantify short‐term repeatability of the W2 scintillator.

#### 2.2.4 Dose‐response linearity

The dose‐response linearity of the W2 was assessed in the reference‐like setup for 6 MV and 6 MV FFF beams. Triggered and manual data collection was performed for MU values ranging from 10 to 1000 MU (approximately 0.065–6.5 Gy). The maximum dose rate was used for each energy. The same measurements were also performed with a Farmer‐type ion chamber to verify the dose linearity of the linac. Root‐mean‐square (RMS) deviations from expected readings were calculated for each series of measurements, following Carrasco et al.^6^

#### 2.2.5 Angular dependence

Angular dependence was assessed using a spherical Lucite phantom (Lucy Phantom, Standard Imaging, Middleton, WI). The W2 scintillator was placed in the center of the phantom and precisely aligned to isocenter using cone beam CT. To measure axial dependence, the W2 was oriented parallel to the gantry rotation axis (perpendicular to the beam axis), and the gantry was rotated to vary axial incidence (gantry angles 15–345° in 30° increments). To measure polar dependence, the W2 was oriented vertically (parallel to couch rotation axis), and the gantry was rotated to vary the polar incidence (gantry angles 0–165° in 15° increments). The measurement at 90° corresponds to perpendicular incidence. The RMS values for angular dependence were calculated using the percent difference with respect to the measurements at gantry angle 15 and 90° for axial and polar dependence, respectively.

#### 2.2.6 Temperature dependence

The temperature dependence of the W2 detector was measured in the reference‐like setup. Measurements were collected with the 6 MV FFF beam. The water temperature was increased from 17.8 to 39.2°C in increments of roughly 5°C. Four readings of 100 MU were collected at each temperature, acquired at intervals of 1 min to determine if any short‐term signal drift occurred after the temperature changed. A linear fit of the average readings at each temperature provided the percent change in W2 response per unit°C.

#### 2.2.7 Dose‐per‐pulse dependence

The dose‐per‐pulse dependence of the W2 was measured with the detector mounted in‐air on a small post, aligned perpendicular to the beam on the central axis. A cylindrical build‐up cap composed of rolled‐up bolus was placed over the detector to put it at a depth of 1.4 cm (*d*_max_) in a 5 × 5 cm^2^ field. The dose‐per‐pulse was varied by changing the table height to achieve different source‐to‐detector distances of 80, 90, 100, 110, 120, and 135 cm. Deviations in detector response from the inverse square law were attributed to dose‐per‐pulse dependence. The RMS deviation for dose‐per‐pulse dependence was calculated using the deviations in detector response from the inverse square law.

#### 2.2.8 MU repetition rate dependence

The dependence of the W2 response on MU repetition rate was measured in the reference‐like setup for 6 MV and 6 MV FFF. Manual data collection was performed for MU repetition rates of 5–600 MU/min for 6 MV, and 400–1400 MU/min for 6 MV FFF. RMS deviation was calculated using the percent differences with respect to the maximum dose rate. Triggered data collection was not used because triggering became unreliable for very low dose rates.

### 2.3 Small field profile measurements

Next, we studied the best practices and performance of the W2 scintillator for profile measurements in a scanning water tank. First, we optimized scan acquisition parameters and explored the effect of the CLR correction on the measured scans. With the scanning parameters optimized, we acquired a final set of scans and compared them to data acquired with a scanning diode detector.

The W2 was oriented perpendicular to the central axis of the beam for all scanning measurements. The manufacturer states that the effective point of measurement is in the center of the cylindrical volume of the detector and 1.3 mm from the tip. We positioned the W2 in the same manner as an ion chamber with the cylindrical body of the detector splitting the water surface. The inline and crossline position of the detector was initially set with the light field, then fine‐tuned with the automated central‐axis correction tool in the scanning software. The W2 fiber was connected to an IBA scanning electrometer via the Standard Imaging MAX‐SD, which applies the CLR and Gain to raw measurements before passing the signal to the scanning electrometer.

#### 2.3.1 Scan technique optimization

Two different apertures were used to investigate scanning techniques for the W2: 1 × 1‐cm^2^ MLC‐shaped field and 4‐mm cone. For each of these apertures, profiles were obtained in continuous scanning mode (speeds of 0.3–1.5 cm/s) and step‐by‐step mode (0.2 s, 0.5 s, 1 s, 2 s integration time). Each profile for a given aperture was compared to the step‐by‐step measurement with 2 s integration time, which we expected to be the least noisy. Profiles were normalized on the central axis and compared using gamma analysis. We used the following criteria: 0.5%/0.5 mm, 1.0%/0.5 mm, 2.0%/0.5 mm, and 1%/1 mm. All analysis used global dose difference without a threshold dose (all points were included).

#### 2.3.2 Effect of CLR on profile measurements

The 4‐mm cone field was scanned multiple times with the W2 software configured to use each of the four CLR measurement methods to assess the effect of CLR on profile measurements. All scans were acquired in step‐by‐step mode with an integration time of 1 s (as deemed appropriate from the scan technique optimization results). Each profile was compared to the measurement acquired with the “5.5 × 10 cm rectangle method.” Profiles were compared using gamma analysis using the same criteria described above.

#### 2.3.3 Profile intercomparisons with IBA RAZOR detector

A final set of inline and crossline profiles was measured for a 1 × 1 MLC‐shaped field and 4‐mm cone. All scans were acquired in step‐by‐step mode with an integration time of 1 s. The CLR measured using the “5.5 × 10 cm rectangle method” was applied. These profiles were compared to data acquired with an IBA RAZOR diode (IBA Dosimetry, Schwarzenbruck, Germany). The diode measurements were also acquired in step‐by‐step mode with an integration time of 1 s. The profiles were compared using gamma analysis using the same criteria described above. For each pair of profiles, we also compared the full‐width half‐maximum and penumbra widths.

### 2.4 Output factor measurements

#### 2.4.1 MLC output factors

MLC output factors were measured in reference‐like setup. Measurements were performed using the W2 at 6 MV FFF for MLC‐defined field sizes of 1 × 1 cm, 2 × 2 cm, and 3 × 3 cm with the jaws at 20 × 20 cm. Output factors were normalized to a 10 × 10 cm field defined by the jaws. Measurements of 200 MU were made with triggered data collection. The output factors measured with the W2 were compared to RAZOR diode‐measured output factors. The RAZOR output factors are relative to a 10 × 10 cm jaw‐defined field and were fully corrected using the guidance and correction factors provided in TRS‐483.^17^

#### 2.4.2 Cone output factors

Radiosurgery cone output factors were measured in an SAD geometry (95 cm SSD, 5 cm depth). Measurements were obtained for the 4, 5, 7.5, 10, 12.5, 15, and 17.5 mm diameter cones for 6 MV FFF. Output factors were normalized to a 10 × 10 cm field defined by the jaws. The output factors measured with the W2 were compared to RAZOR diode‐measured output factors. The RAZOR diode output factors were corrected using TRS‐483.

#### 2.4.3 Effect of CLR on output factor measurements

Once data acquisition was completed, we used the raw blue and green channel data for each measurement to recalculate the output factors using Equation 3 with the four different CLR correction factors. This allowed us to quantify the effect of the CLR on output factor measurements.

### 2.5 Composite treatment plans

Finally, the dose at isocenter for seven radiosurgery plans was measured with the W2 using a Lucy phantom. Table 1 shows the characteristics of the seven radiosurgery plans selected for measurement. The plans were originally generated in Eclipse (Varian Medical Systems, Palo Alto, CA). The cone‐based plans use five or more static arcs at different couch angles, while the MLC‐based plans use five or more dynamic conformal arcs at different couch angles. The plans were recalculated on a CT of the Lucy phantom with isocenter placed at the center of the W2 detector volume.

**TABLE 1:** Characteristics of radiosurgery plans

Plan Number	Indication	Target volume (cc)	Equivalent sphere diameter (cm)	Aperture definition
1	Brain metastasis	3.11	1.8	MLCs
2	Brain metastasis	0.15	0.7	7.5‐, 10‐mm cones
3	Brain metastasis	0.52	1.0	MLCs
4	Brain metastasis	0.06	0.5	5‐, 7.5‐mm cones
5	Brain metastasis	3.8	1.9	MLCs
6	Brain metastasis	0.06	0.5	5‐, 7.5‐mm cones
7	Trigeminal neuralgia	N/A	N/A	4‐mm cone

**FIGURE 2:**
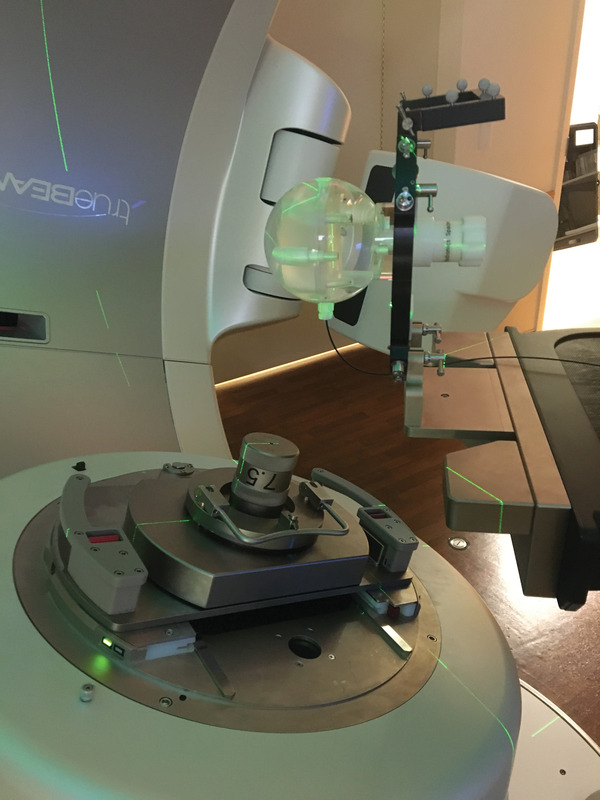
The measurement geometry used for radiosurgery plans

Figure 2 shows the measurement geometry. The Lucy phantom was mounted over the end of the TrueBeam IGRT couch in a radiosurgery frame. The W2 entered the phantom from the bottom such that it was oriented parallel to couch rotation axis. In this orientation, couch rotation of the phantom results in axial rotation of the detector, and gantry rotations result in different polar incidence angles. Before delivering the treatment plans, the Lucy phantom was positioned using cone‐beam CT.

The treatment plans were delivered in TrueBeam Treatment Mode as a QA patient. The raw blue and green channel readings (in pC) were recorded for each arc. The raw readings were converted to dose via a spreadsheet using the CLR and Gain generated with the 5.5 × 10 cm rectangle method and Equation 3. The dose delivered for the full plan was the sum of the dose measurements for all arcs for a given plan. This plan dose was compared to the dose calculation in Eclipse to determine agreement between W2 measurement and dose calculation.

#### 2.5.1 Effect of CLR on measured dose

The use of a spreadsheet to calculate the dose from raw channel values allowed us to easily assess the impact of different CLR measurement methods on the dose measurements. The CLR and Gain values from all four measurement methods were used to recompute the measured dose, and these doses were compared to dose calculated in Eclipse.

#### 2.5.2 Effect of polar angle dependence on measured dose

We chose the detector orientation shown in Figure 2 so that the W2 response would be independent of couch angle. In this orientation, the W2 exhibits polar angle dependence during gantry rotation. The polar angle dependence results in higher readings when delivering from the tip‐side of the detector and lower readings when delivering from the stem side. Based on the results of our angular dependence testing, we estimated that the polar angle correction would approximately cancel out for the types of arcs we use for radiosurgery and would not need to be accounted for during routine use of the system for patient‐specific QA. To test this assumption, we calculated the measured dose with and without employing a polar angle correction based on the measured polar angle dependence to see the impact of the polar angle dependence for clinical plans. The polar angle dependence measurements from section B.5 were used to calculate a polar angle correction for each arc by integrating the polar angle dependence data over the range of angles from the gantry start position to the stop position. The corrected doses for each arc were summed to determine a polar‐angle‐corrected plan dose, which was compared to the calculated dose from Eclipse.

## 3 RESULTS AND DISCUSSION

### 3.1 LR

Table 2 shows the CLR and Gain values measured using each of the four techniques for 6 MV FFF. Excluding the “2 × 3 cm rectangle method”, the CLR and Gain values of the other three methods varied by less than 0.25% and 1%, respectively. The CLR and Gain values for the “2 × 3 cm rectangle method” differed from the others by more than 2%.

**TABLE 2:** Čerenkov light ratio (CLR) and gain values measured using the four CLR measurement techniques for 6 MV FFF

Measurement technique	CLR	Gain
10 × 1 cm rectangle method	0.970	2.397
5.5 × 10 cm rectangle method	0.972	2.398
3 × 5 cm rectangle method	0.970	2.375
2 × 3 cm rectangle method	0.946	2.344

### 3.2 General characterization

Table 3 is a summary of the general characterization testing and includes results from Carrasco et al.^6^ and Galavis et al.^12^ for comparison.

**TABLE 3:** Summary of general characterization testing, with results from Carrasco et al. and Galavis et al. for comparison

Test	Our result (W2 detector)	Carrasco et al. 2015 (W1 detector)	Galavis et al. 2019 (W2 detector)
Energy dependence	Max. deviation 2.3%	Negligible (k = 2)	N/A
Settling behavior	*σ*_Green_ = 0.03 pC *σ*_Blue_ = 0.03 pC RMS_Green_ = 0.20% RMS_Blue_ = 0.8%	N/A	*σ*_Green_ = 0.04 pC *σ*_Blue_ = 0.03 pC
Short‐term repeatability	*σ*_100 MU, triggered_ = 0.09% *σ*_100 MU, manual_ = 0.18% *σ*_20 MU, manual_ = 0.21%	*σ*_100 MU, triggered_ = 0.10% *σ*_100 MU, manual_ = 0.40% *σ*_20 MU, manual_ = 2.22%	N/A
Dose‐response linearity	RMS_6MV_ = 0.11% RMS_6 MV FFF_ = 0.13%	RMS = 0.61%	“dose linearity readings were within 0.05%”
Angular dependence (axial)	RMS = 0.24%	RMS = 0.21%	N/A
Angular dependence (polar)	RMS = 1.48%	N/A	N/A
Temperature dependence	−0.180%°C^–1^	−0.225%°C^–1^	−0.170%°C^–1^
Dose per pulse dependence	RMS = 0.35%	RMS = 0.38%	N/A
Repetition rate dependence	RMS_6MV_ = 0.66% RMS_6 MV FFF_ = 0.19%	RMS = 0.53%	“linear above 200 MU/min”

*Note*: Our results are for 6 MV FFF unless otherwise stated. Carrasco combined measurements for 6 and 15 MV photons when reporting *σ* and RMS values. Galavis’ results are for 6 MV.

Abbreviation: RMS, root‐mean‐square.

#### 3.2.1 Energy dependence

Among all four energies, the CLR values varied by as much a 1.0% (range 0.977‐0.987) and the Gain values varied by as much as 2.4% (range 2.269‐2.324). The “6 MV FFF Cal” column of Table 4 shows the percent difference between the doses measured with the W2 scintillator calibrated at 6 MV FFF and the doses measured with the ion chamber. We found that using the 6 MV FFF calibration to measure dose for a different beam energy resulted in a measured dose that differed from expectations by more than a *k* = 2 uncertainty limit. We repeated this experiment with the W2 software configured to use the CLR and Gain for our other beam energies to see if there was something anomalous about the 6 MV FFF calibration. We obtained the same result: The CLR and Gain for each energy produced an accurate dose measurement for that energy, but not for the other energies (Table 4). These results differ from the W1 measurements performed by Carrasco et al., who found a negligible energy dependence for 6 and 15 MV x‐ray beams (‐0.2 ± 0.6 %). We conclude that the W2 exhibits a small energy dependence (maximum deviation 2.3%) and should be calibrated for each unique energy for applications that require greater accuracy.

**TABLE 4:** The percent difference between the dose measured with the W2 scintillator and the dose measured with the ion chamber

	6 MV FFF Cal	6 MV Cal	10 MV FFF Cal	10 MV Cal
**6 MV FFF**	0.0 ± 0.6	−1.3 ± 0.2	−1.8 ± 0.2	−2.3 ± 0.2
**6 MV**	0.9 ± 0.3	0.0 ± 0.3	−0.7 ± 0.2	−1.0 ± 0.4
**10 MV FFF**	1.6 ± 0.3	0.5 ± 0.2	0.0 ± 0.1	−0.5 ± 0.2
**10 MV**	1.9 ± 0.2	0.6 ± 0.2	0.2 ± 0.1	−0.1 ± 0.2

*Note*: Each row represents output measurements for a given beam energy using the four different calibrations (columns). The uncertainty is expressed with *k* = 2.

#### 3.2.2 Detector settling behavior

The detector settling behavior for the W2 is small. The relative deviation of the blue and green channels over the course of 15 min was 0.075% and 0.204%, respectively. Of note, the green channel exhibited a 1% drift over the first 90 s that settled thereafter. Excluding the first 90 s of data collection, the relative deviation of the blue and green channels was 0.067% and 0.161%, respectively. The higher relative deviation of the green channel reflects the lower signal for this channel relative to the blue channel.

#### 3.2.3 Short‐term repeatability

The relative deviation of the short‐term repeatability measurements for the 6 MV FFF beam were 0.18%, 0.21% and 0.09% for 100 MU manual, 20 MU manual and 100 MU triggered, respectively (Table 3). The relative deviations of the 6 MV beam measurements were similar (0.15%, 0.30%, and 0.12%, respectively). The short‐term repeatability relative deviations measured by Carrasco et al. for the W1 detector were similar except at 20 MU manual, for which the performance of the W2 was much better (2.22% for W1 vs. 0.30% at 6 MV and 0.21% at 6 MV FFF for W2).

#### 3.2.4 Dose‐response linearity

The response of the W2 scintillator was linear with MU. Using the W2 in triggered mode produced a similar RMS deviation as an ion chamber. For 6 MV, the RMS deviations were 0.36%, 0.11% and 0.08% for the manual W2, triggered W2 and ion chamber measurements, respectively. For 6 MV FFF, the RMS deviations were 0.28%, 0.13% and 0.13%, respectively.

The RMS value of 0.11% measured for 6 MV triggered mode in this work is much lower than the 0.64% measured with the W1 by Carrasco et al.^6^ The larger value reported by Carrasco et al. was heavily influenced by the lowest dose reading, for which the deviation was almost 2%. The largest deviation we measured was 0.85% (6 MV, manual). Galavis et al.^12^ did not report an RMS value for dose‐response linearity but stated that dose linearity measurements with the W2 in a 6 MV beam were within 0.05% over a range of 1 to 1000 MU.

#### 3.2.5 Angular dependence

**FIGURE 3:**
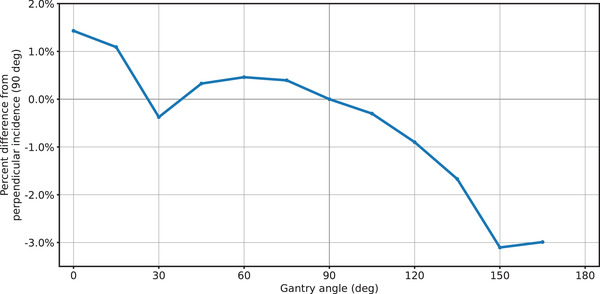
Polar angle dependence of the W2 scintillator, normalized at perpendicular incidence

The axial angular dependence of the W2 was small. The maximum axial angular dependence of the detector was 0.43% with an RMS value of 0.24%. This agrees well with the axial angular dependence reported with the W1 by Carrasco (RMS = 0.21%). The W2 scintillator demonstrated a greater dependency on polar angle with an RMS value of 1.48% and a maximum deviation of 3.1%. These values are larger than the 1% maximum polar dependence demonstrated by Dimitriadis et al.^10^ with the W1 scintillator. The polar angle dependence is plotted in Figure 3.

The presence of a polar angle dependence is perhaps to be expected given the physics of Čerenkov radiation. Čerenkov light is emitted at a characteristic angle relative to the incident radiation beam. The angle depends on the relativistic speed of the secondary electrons and the index of refraction of the medium. Changing the polar angle of the beam will change the angle between the Čerenkov light emission and the orientation of the fiber.^18^, ^19^ Changing polar angle also changes the amount of fiber in the radiation field, so the polar angle effect is a combination of multiple factors. In general, we saw in higher readings when delivering radiation from the tip‐side of the detector and lower readings when delivering from the stem side. This is consistent with the findings of Jang et al.,^19^ who studied a Čerenkov light‐based radiation measurement system and found a higher signal when delivering radiation from the tip‐side of the detector. Changing polar angle may invalidate the CLR correction by changing the relative amount of Čerenkov light reaching the photodiode for a given unit of dose. Indeed, Simiele et al.^11^ measured CLR over a range of polar angles and found that it varied by 3.5% between 0‐ and 90‐degree incidence. We advise users of the W2 to carefully characterize polar dependence when it will be relevant in a measurement scenario.

#### 3.2.6 Temperature dependence

The temperature dependence of the W2 was linear over the range of 17.8‐39.2°C. The rate of change was ‐0.181%/°C. This is similar to the value measured by Galavis et al.^12^ (‐0.170%/°C) The relative deviation among measurements acquired at the same temperature over the course of several minutes did not exceed 0.05%, suggesting that the detector equilibrates to changes in temperature very quickly.

#### 3.2.7 Dose‐per‐pulse dependence

The detector response displayed minimal deviations from the inverse square law with an RMS of 0.35%, as displayed in Table 3. This result agreed well with the RMS value of 0.38% measured for the W1 by Carrasco et al.^6^

#### 3.2.8 MU repetition rate dependence

RMS deviations of 0.66 and 0.19% were calculated for measurements performed with the 6 MV and 6 MV FFF beams, respectively. These values agree well with measurements performed by Carrasco et al.^6^ with the W1 scintillator (Table 3). For repetition rates greater than 20 MU/min, the dose deviations relative to the maximum dose rate were less than 0.5%. Below 20 MU/min, a maximum deviation of 2% was found for a repetition rate of 10 MU/min with the 6 MV beam. Galavis et al.^12^ reported that MU repetition rate response of the W2 was linear above 200 MU/min, but measurements at 100 MU/min and lower had large standard deviations.

### 3.3 Small field profile measurements

#### 3.3.1 Scan technique optimization

**FIGURE 4:**
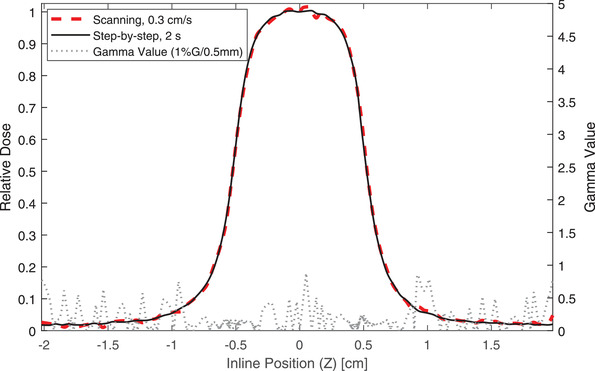
Inline profile measurements of a 1 × 1 cm^2^ MLC‐shaped field. Both measurements were acquired with the W2 plastic scintillator using different scan settings (black = step‐by‐step measurement with 2 s integration time, red = scanning measurement with a scanning speed of 0.3 cm/s). The gamma values from a 1D gamma analysis are plotted as well (1% global dose difference, 0.5 mm distance‐to‐agreement)

Tables 5 and 6 contain results from the scanning technique comparisons. The step‐by‐step profile acquired with 2 s integration was used as the gold standard. In step‐by‐step mode, the minimum gamma passing rates at 0.5%G/0.5 mm, 1%G/0.5 mm, 2%G/0.5 mm, and 1%G/1 mm were 97%, 99.5%, 100%, and 100%, respectively. Scans with shorter integration times have slightly noisier appearance. In scanning mode, the minimum gamma passing rates at 0.5%G/0.5 mm, 1%G/0.5 mm, 2%G/0.5 mm, and 1%G/1 mm were 85.1%, 93.1%, 97.5, and 98.5%, respectively. The scans became progressively noisier as the scan speed increased. Figure 4 shows an example comparison for the 0.3 cm/s scan versus the 2 s integration step‐by‐step measurement. The presence of noise in the scanning measurement is visible in the 0.3 cm/s profile.

**TABLE 5:** Gamma analysis results comparing different scanning techniques for relative profile measurements of a 1 × 1 cm MLC‐shaped field

Scantechnique	Integration time (s)	Speed (cm/s)	0.5%G/0.5 mm	1%G/0.5 mm	2%G/0.5mm	1%G/1 mm
Scanning		0.3	96.9	100	100	100
Scanning		0.6	95.7	99.4	100	100
Scanning		0.9	91.2	97.2	99.5	100
Scanning		1.2	85.1	93.1	99.2	98.5
Scanning		1.5	87.3	95.1	97.5	100
Step by Step	0.2		97.8	99.5	100	100
Step by Step	0.5		97	99.8	100	100
Step by Step	1		99	100	100	100
Step by Step	2		*100*	*100*	*100*	*100*

*Note*: The step‐by‐step profile acquired with 2 s integration was used as the gold standard.

**TABLE 6:** Gamma analysis results comparing different scanning techniques for relative profile measurements of a 4‐mm radiosurgery cone

Scantechnique	Integration time (s)	Speed (cm/s)	0.5%G/0.5 mm	1%G/0.5 mm	2%G/0.5 mm	1%G/1 mm
Scanning		0.3	99.8	100	100	100
Scanning		1.5	89	96.6	99.4	100
Step by step	0.2		99.2	100	100	100
Step by step	0.5		99	100	100	100
Step by step	1		100	100	100	100
Step by step	2		*100*	*100*	*100*	*100*

*Note*: The step‐by‐step profile acquired with 2 s integration was used as the gold standard.

For high quality measurements that will be used for beam modeling, we recommend step‐by‐step scanning with an integration time of at least 1 s at our institution. Shorter integration times result in greater noise and declining gamma passing rates relative to the ideal scenario. The use of the detector in scanning mode resulted in noticeable noise in the profiles, so we do not recommend the use of the W2 in scanning mode for beam data acquisition. That said, 0.3 cm/s was the slowest speed available with our scanning system. It is possible that slower scanning speeds, if available, may result in acceptable quality measurements.

For scenarios in which the detector will be used for constancy comparisons (for example, annual QA), it may be possible to use shorter integration times or scanning mode. This will depend upon the use case and the acceptable amount of noise in the measurement.

#### 3.3.2 Effect of CLR on profile measurements

Table 7 contains results from the comparisons of scans acquired with different CLR and Gain values applied. The profile acquired with the “5.5 × 10 cm rectangle method” CLR was used as the gold standard. The minimum gamma passing rates at 0.5%G/0.5 mm, 1%G/0.5 mm, 2%G/0.5 mm, and 1%G/1 mm were 98.8%, 100%, 100%, and 100%, respectively. These high gamma passing rates indicate that relative profile measurements are not dependent upon the CLR.

**TABLE 7:** Gamma analysis results comparing four Čerenkov light ratio (CLR) measurement methods for relative profile measurements

CLR	Scan direction	0.5%G/0.5 mm	1%G/0.5 mm	2%G/0.5 mm	1%G/1 mm
5.5 × 10	Crossline	*100*	*100*	*100*	*100*
	Inline	*100*	*100*	*100*	*100*
3 × 5	Crossline	100	100	100	100
	Inline	99.2	100	100	100
2 × 3	Crossline	99.4	100	100	100
	Inline	100	100	100	100
10 × 1	Crossline	98.8	100	100	100
	Inline	99.5	100	100	100

*Note*: Gamma analysis results for the W2 comparing four CLR measurement methods for the 6FFF 4 mm cone field. The profiles acquired with the “5.5 × 10 cm rectangle method” are used as the gold standard.

#### 3.3.3 Profile intercomparisons with IBA RAZOR detector

**FIGURE 5:**
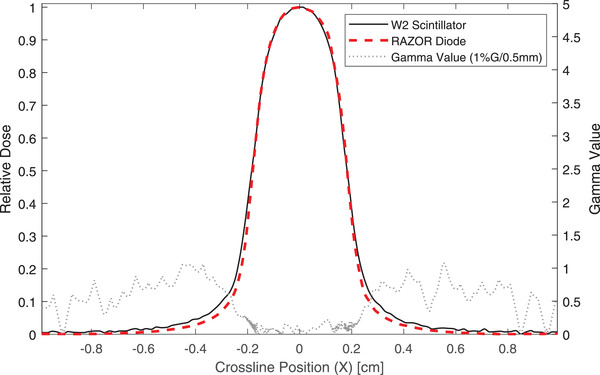
Crossline profile measurements of a 4‐mm cone‐shaped field. The measurements were acquired with the W2 plastic scintillator (black) and an IBA RAZOR diode (red). The gamma values from a 1D gamma analysis are plotted as well (1% global dose difference, 0.5 mm distance‐to‐agreement)

Table 8 contains results from the comparisons of the W2 detector with the IBA RAZOR diode detector. The minimum gamma passing rates at 0.5%G/0.5 mm, 1%G/0.5 mm, 2%G/0.5 mm, and 1%G/1 mm are 64.3%, 96.1%, 100%, and 99.7%. The failing points in the profiles are largely in the tails and the out‐of‐field region. An example of this is shown in Figure 5. If a low‐dose threshold of 10% had been applied to the analysis, the pass rate would have been close to 100% for all comparisons. Full‐width half‐maximum and penumbra widths agreed to within 0.1 mm.

**TABLE 8:** Gamma analysis results for the comparison of the W2 detector with the IBA RAZOR diode

Aperture	Scan direction	0.5%G/0.5 mm	1%G/0.5 mm	2%G/0.5 mm	1%G/1 mm
1 × 1 MLC	Crossline	93.6	100	100	100
	Inline	87.9	99.5	100	99.7
4 mm Cone	Crossline	90.3	99.1	100	100
	Inline	64.3	96.1	100	100

The differences seen in the profiles represent real differences between the detectors, which have different sizes in the beams‐eye‐view and different response characteristics in small fields.^17^, ^20^ The profiles measured with the W2 have slightly narrower shoulders and wider tails compared to the RAZOR diode. These differences are typical of detector volume averaging effects, which are expected because the diameter of the W2 in the beams‐eye‐view is approximately double that of the RAZOR diode.^17^ In addition to differences due to volume averaging effects, the measured dose in the tails and out‐of‐field region is lower for the RAZOR diode compared to the W2. This cannot be accounted for by volume averaging but may be linked to detector‐specific small‐field behaviors. Francescon et al.^20^ performed Monte Carlo simulations of many detectors for small field dosimetry, including the W1 plastic scintillator and an IBA diode. The simulations showed that all diodes, including the IBA diode, are expected to under‐respond relative to the W1 in the out‐of‐field region by approximately 5% of the local dose at the edge of the field and as much as 15% at twice the radius of the field. The relative profiles that we measured with W2 and RAZOR diode differ in a manner that is qualitatively similar to the behavior predicted by Monte Carlo for detectors of similar designs.

Studies that have compared the W2 to other measurement devices for relative profile measurements have generally found good agreement among devices. Galavis et al.^12^ used the W2 and GafChromic EBT3 film to measure relative profiles for a 6 MV 1 × 1 cm^2^ field at three depths. They reported that the W2 and film agreed to within 0.5% for all measurements. Rudek et al.^13^ intercompared the W2, a PTW microdiamond detector and Gafchromic EBT3 film for measurement of relative profiles for the Gamma Knife. They did not quantify the extent to which the relative profiles agreed with one another but did report how well the measured full‐width half‐maximum and penumbra width agreed with the treatment planning system. In general, the W2, microdiamond and film showed similarly high levels of agreement with the Gamma Plan treatment planning system. One exception was that the W2 systematically measured the penumbra of the z‐axis profiles to be larger than the treatment planning system and other measurement tools. The maximum difference between the W2 and treatment planning system was 0.31 mm, less than the tolerance value of 1 mm.

### 3.4 Output factor measurements

#### 3.4.1 MLC output factors

Table 9 shows the output factors for MLC‐shaped fields measured with the W2 compared with the corrected RAZOR output factors. Output factors for the two detectors agree to within 1.8%. Galavis et al.^12^ measured output factors for MLC‐shaped fields with the W1 and W2 scintillators. They found that output factors measured with the two devices agreed to within 1% except for the smallest field size of 0.5 × 0.5 cm^2^, for which the difference was 5%.

**TABLE 9:** MLC and cone output factors measured with the W2 and the RAZOR diode

MLC‐defined field size (cm x cm)	Cone diameter (mm)	W2 output factor	RAZOR output factor	% Difference
1 × 1	–	0.755	0.742	1.8%
2 × 2	–	0.832	0.820	1.5%
3 × 3	–	0.872	0.861	1.3%
–	4	0.581	0.572	1.5%
–	5	0.637	0.628	1.4%
–	7.5	0.728	0.718	1.3%
–	10	0.784	0.774	1.3%
–	12.5	0.82	0.809	1.3%
–	15	0.846	0.834	1.5%
–	17.5	0.864	0.851	1.6%

#### 3.4.2 Cone output factors

Table 9 also shows the output factors for cone‐shaped fields measured with the W2 compared with the RAZOR diode output factors. Output factors for the two detectors agree to within 1.6% for all cone‐shaped fields. The percent differences do not vary by more than 0.3% over the range of field sizes. The results for MLCs and cones are remarkably consistent. There appears to be a small, on average 1.5%, difference in output factors between the two detectors across all fields measured in this work.

#### 3.4.3 Effect of CLR on output factor measurements

Table 10 shows the W2 output factors for cone‐shaped fields calculated with CLR values from the four measurement techniques. Relative to using the “5.5 × 10 cm rectangle” CLR, the “10 × 1 cm rectangle” and “3 × 5 cm rectangle” CLRs result in negligible changes in the output factors (maximum −0.1%). The “2 × 3 cm rectangle” CLR has a larger effect on the output factors (maximum −1.0%).

**TABLE 10:** Cone output factors computed with four different Čerenkov light ratio (CLR) measurement methods

	RAZOR	5.5 × 10		3 × 5		2 × 3		10 × 1	
Cone diameter (mm)	OF	OF	% Diff	OF	% Diff	OF	% Diff	OF	% Diff
4	0.572	0.581	1.5%	0.580	1.4%	0.575	0.5%	0.580	1.4%
5	0.628	0.637	1.4%	0.636	1.3%	0.631	0.4%	0.636	1.3%
7.5	0.718	0.728	1.3%	0.727	1.2%	0.721	0.4%	0.727	1.2%
10	0.774	0.784	1.3%	0.784	1.2%	0.777	0.4%	0.783	1.2%
12.5	0.809	0.820	1.3%	0.819	1.3%	0.813	0.4%	0.819	1.2%
15	0.834	0.846	1.5%	0.846	1.4%	0.839	0.6%	0.846	1.4%
17.5	0.851	0.864	1.6%	0.864	1.5%	0.857	0.7%	0.864	1.5%

Recall that CLRs are generated with maximum and minimum fiber in‐field geometries. The 10 × 10 cm reference field measurement used to compute the output factors has length of fiber in‐field that exceeds the maximum used for the “2 × 3 cm rectangle” and “3 × 5 cm rectangle” CLRs. Therefore, these CLRs may not be reliable for this measurement scenario. The “5.5 × 10 cm rectangle” CLR and the “10 × 1 cm rectangle” are a more appropriate choice if the normalization field size is 10 × 10 cm^2^.

### 3.5 Composite treatment plans

Table 11 shows the difference between the measured and calculated dose for each plan. Overall, the agreement between measurements and dose calculations was better than or equal to 2.18% for all plans.

**TABLE 11:** The effect of Čerenkov light ratio (CLR) on the composite treatment plans

		Difference between measurement and calculation (%)			
Plan Number	Equivalent squarediameter (cm)	5.5 × 10	3 × 5	2 × 3	10 × 1 cm
1	1.8	0.29%	0.29%	0.22%	0.29%
2	0.7	1.15%	1.12%	0.72%	1.10%
3	1.0	2.18%	2.16%	1.92%	2.15%
4	0.5	0.56%	0.53%	0.21%	0.51%
5	1.9	0.01%	0.01%	‐0.07%	0.00%
6	0.5	0.82%	0.79%	0.47%	0.78%
7	N/A	1.19%	1.15%	0.64%	1.13%

#### 3.5.1 Effect of CLR on measured dose

Table 11 also shows the effect of CLR measurement method on the composite dose measurements. Relative to using the “5.5 × 10 cm rectangle” CLR, the “10 × 1 cm rectangle” and “3 × 5 cm rectangle” CLRs result in negligible changes in the measured dose (maximum −0.06% for plan 7). The “2 × 3 cm rectangle” CLR has a larger effect on the measured dose (maximum −0.55% for plan 7, relative to the “5.5 × 10 cm rectangle” CLR). During commissioning of the treatment planning system, our clinic established that the treatment planning system has an accuracy of 2% or better for point dose measurements at isocenter. The effect of CLR (0.55% maximum) is smaller than the established accuracy of the treatment planning system and can be considered negligible.

#### 3.5.2 Effect of polar angle dependence on measured dose

A total of 38 arcs were delivered over the seven test plans. The minimum, average, and maximum polar angle correction factor was 0.971, 0.996, and 1.005, respectively. Of the 38 arcs that were delivered, 36 arcs had a polar angle correction factor that was less than 1% (0.99–1.01). Table 12 shows the difference between the measured and calculated dose for each plan, with and without the polar angle correction applied. The average change in measured plan dose was −0.4% (range −0.71% to +0.05%). This shows the application of the polar angle correction generally decreases the measured dose. Overall, there was a modest improvement in agreement with the calculated dose after the polar angle correction was applied. Agreement improved in five of the seven plans, and the average difference changed from 0.89% without the correction applied to 0.47% with the correction applied.

**TABLE 12:** The effect of polar angle correction on the composite treatment plans

	Equivalent square diameter (cm)	Difference between measurement and calculation (%)	
Plan number		No polar angle correction	With polar angle correction
1	1.8	0.29%	−0.13%
2	0.7	1.15%	0.92%
3	1.0	2.18%	1.55%
4	0.5	0.56%	−0.16%
5	1.9	0.01%	−0.51%
6	0.5	0.82%	0.41%
7	N/A	1.19%	1.24%

We hypothesized that the polar angle correction factor would approximately cancel out, and this turned out to be true. Although the polar angle correction reached 2.9% for one of the individual arcs, the overall effect of correcting for polar angle for a plan was never larger than 0.71%. Correcting for polar angle effects may be of value for commissioning a radiosurgery system if utmost accuracy is desired. The polar angle correction calculation can be automated using a spreadsheet for routine use of the W2 in patient‐specific QA. Alternatively, polar angle effects can be ignored during routine use of the W2 if the effect is accounted for when determining tolerance values for testing.

## 4 CONCLUSION

The W2 exhibited dosimetric characteristics that are suitable for radiosurgery field measurements. The detector exhibits a dependence upon energy, polar angle, and temperature. Dependence upon energy can be handled by measuring a separate CLR and Gain for each beam energy. Polar angle dependence can be managed by applying an angle‐dependent correction or restricting use of the detector to a single polar angle. Temperature dependence is relatively small per degree Celsius but may need to be addressed in situations where absolute dose measurements are required.

The W2 detector performed well in the types of measurements that are required for commissioning a treatment delivery system for radiosurgery. Experiments with scanning measurements showed no dependence on CLR, and measurements done with the W2 and RAZOR diode showed good agreement. Differences were largely in the tails and the out‐of‐field regions of the profiles. In measurements of clinical radiosurgery plans, the W2 showed good agreement with the treatment planning system.

## CONFLICT OF INTEREST

The authors have no conflict of interest to disclose.

## AUTHOR CONTRIBUTIONS

All four authors made substantial contributions to the conception and design of the research project. This included the acquisition, analysis, and interpretation of data for the manuscript. Dustin and Jessica were involved in drafting the work. All four authors revised the work critically for important intellectual content. All four authors gave final approval of the version to be published and agree to be accountable for all aspects of the work in ensuring that questions related to the accuracy or integrity of any part of the work are appropriately investigated and resolved.
